# Brief hospitalizations of elderly patients

**DOI:** 10.1186/1757-7241-20-S2-P6

**Published:** 2012-04-16

**Authors:** Sofie Strømgaard, Søren Wistisen Rasmussen, Thomas Andersen Schmidt

**Affiliations:** 1 The Emergency Department, Holbæk University Hospital, Denmark

## Background

Crowded departments are a common problem in Danish hospitals, especially in departments of internal medicine, where a large proportion of the patients are elderly. We therefore chose to investigate the number and character of hospitalizations of elderly patients with a duration of less than 24 hours, as such short admissions could indicate that the patients had not been severely ill and that it might have been possible in these cases to avoid hospitalization.

## Methods

Medical records were examined to determine the number of patients aged 75 or more who passed through the emergency department over a period of two months, and the proportion of those patients who were discharged after less than 24 hours. The reasons for the hospitalization, the diagnoses and the treatment given were noted.

## Results

Twenty-four percent of the older patients were discharged after less than 24 hours. Of these, 40 % were discharged from the emergency department. The most common problems leading to hospitalization were change in contact or level of consciousness, focal neurological change, red, swollen or painful leg conditions, dyspnoea, suspected parenchyma surgical disease and problems with the urinary system or catheters. The most common diagnoses given at hospital were chronic cardiovascular disease, bacterial infection, symptoms deriving from bone, muscle or connective tissue, liquid or electrolyte derangement and observation for suspected stroke or transient cerebral ischemia. Eight percent of the patients required telemetry, 27 % received intravenous liquids, 30 % had diagnostic radiology procedures performed and 3 % needed invasive procedures. Other types of treatment given included electrocardiography, laboratory examinations, oxygen supplements, urinary catheterization and medicine administered orally, subcutaneously, as an intramuscular injection or as an inhalation.

## Conclusion

There appears to be a group of patients who cannot be adequately handled with the resources of the primary health care sector, yet who do not belong at the emergency department. Further studies are needed to create a suitable service for these patients, and to improve the continuity of the treatment and the cooperation between hospitals and the primary health care sector.

